# Usefulness of presepsin (sCD14-ST) measurements as a marker for the diagnosis and severity of sepsis in systemic inflammatory response syndrome

**DOI:** 10.1186/cc9834

**Published:** 2011-03-11

**Authors:** T Shozushima, Y Suzuki, T Masuda, G Takahashi, S Endo

**Affiliations:** 1 Iwate Medical University, Morioka, Japan

## Introduction

CD14 is present in macrophage, monocyte, and granulo-cyte cells and their cell membranes, and is said to be responsible for intracellular transduction of endotoxin signals. Its soluble fraction is present in blood and is thought to be produced in association with infections. It is called the soluble CD14 subtype (sCD14-ST), and in the text below it will be referred to by its generic name, presepsin. We have previously reported that presepsin is produced in association with infection and that it is specifically expressed in sepsis. In the present study we developed a new rapid diagnostic method using a chemiluminescent enzyme immunoassay, and it made automated measurements in a shorter time possible.

## Methods

The subjects were 41 inpatients (25 males and 16 females), 62 ± 19 years old, who had been brought to the Critical Care and Emergency Center of Iwate Medical University, and who fulfilled at least two of the diagnostic criteria for systemic inflammatory response syndrome (SIRS) on arrival. Blood specimens were collected a total of six times - that is, on admission, and 12 and 24 hours and 3, 5, and 7 days later - and the presepsin values were measured. The sepsis markers PCT, IL-6, and CRP were also measured for comparison.

## Results

The results of using this method to measure presepsin values in different pathological conditions were: normal, 294.2 ± 121.4 pg/ml; local infection, 721.0 ± 611.3 pg/ml; SIRS, 333.5 ± 130.6 pg/ml; sepsis, 817.9 ± 572.7 pg/ml; and severe sepsis 1,992.9 ± 1,509.2 pg/ml, and the presepsin values were significantly higher in patients with local infection, sepsis, and severe sepsis than in patients who did not have infection as a complication. In a comparative study with other diagnostic markers of sepsis based on ROC curves, the area under the curve (AUC) of presepsin was 0.845, and higher than the AUC of PCT (0.652), CRP (0.815), or IL-6 (0.672).

## Conclusions

In the present study we were able to obtain results similar to those obtained with the conventional ELISA method, and it was possible to diagnose sepsis more rapidly and conveniently by using the immunoassay analyzer. We are currently using the analyzer in a multicenter clinical study, and are in the process of conducting a further clinical dynamics analysis in various pathological conditions. Based on the results of the present study, it appears that presepsin will soon be widely used as a diagnostic marker of sepsis in clinical settings.

